# Medical Education in America

**Published:** 1887-10

**Authors:** 


### MEDICAL EDUCATION IN AMERICA.

The importance of this subject, and its continued disregard, compels us to write very frequently upon it, but we are not without hope that before many years the thoroughness of medical education in America will be equal to that demanded in Europe.

In no other country in the world has the medical profession assumed such formidable proportions as in the United States. In the infant republic of France, one physician is deemed sufficient to care for the physical ills of 4,000 inhabitants; in Germany, there is one doctor to every 2,000 inhabitants, but in our land of light and progress there is one physician to every 600 people, and in our great metropolis there is said to be a doctor and a-half to every tax-payer. A hundred American medical colleges are engaged in the noble occupation of manufacturing doctors. These colleges not only supply a demand according to the usual economic law, but demand a supply, and get it—such as it is. The size of the profession has far more than kept pace with the increase in population, but its respectability has varied inversely with the mass. Granting that some of the world's greatest physicians and surgeons have been produced in America; granting that the country has done its full share in farthering the onward march of medical discovery—still the vast numbers of ignorant quacks who have been licensed under the auspices of the government and tolerated by the community, are no credit to the profession or the nation. Many young men who are too ignorant to succeed in the law, too lazy for manual labor, or too immoral to join the clergy, think that they can gain a comfortable and respectable livelihood in the medical profession, where deception is so easy and ignorance so readily concealed.

Our present system of medical education is largely to be blamed for the unfortunate condition of the profession. The incompetency of law-makers, the ignorance of the people, and the carelessness even to perversity of the more respectable portion of the profession itself, are all to be arraigned. Rural statesmen and Bowery politicians are not competent to make laws regulating the practice of medicine. This should be entrusted to, or at least supervised by, men who understand the true requirements of the profession. If this were done, there would be no more "Dean Buchanans," no more "doctors" turned out from a medical college complete in nine months, having about as much knowledge of their *alma mater* as they acquired in the womb of their *vera mater* during the same length of time.

There once existed "The Central Medical College of Rochester." The faculty in this remarkable institution were the students—the students, the faculty. At the close of a short and comprehensive course of study, the faculty conferred degrees upon themselves, and some are practitioners in our midst to-day. While the church takes an interest in the clergy, and the State educates the teacher, the training of physicians is left to the enterprise of stock companies and private individuals. The seven men who constitute the faculty of many medical colleges are at once proprietors and professors, just as much as "Mr. Squeers" was both principal and proprietor of "Do-the-boys Hall." In other countries, no university is complete without its medical department. Chairs of anatomy and surgery are endowed, as well as those of homiletics or ethics; but in America, there are not a dozen medical schools that have any real connection with a university. The professors are entirely dependent upon the fees of the students for their salaries, and are, therefore, naturally more interested in the quantity than the quality of their graduates. The rapid increase and defective management of medical schools has tended to cause a backward movement of the standard of education. "Over one hundred years ago," says Dr. Roosa, "when the medical department of the University of Pennsylvania was established, it was the intention of the founders to have as liberal and extensive a plan of education as the oldest European universities." A thorough literary and scientific education was a pre-requisite to admission, and only after a three years' course was the degree of Bachelor of Medicine conferred. This was at a time when medical science, compared with that of the present day, was in its infancy. Pathology, histology and a score of subordinate branches of medicine have since come to light. Physiology has been revolutionized by the microscope and modern discoveries in physics. Materia medica has been enriched by the advance in chemistry; but instead of increasing the requirements for admission to our colleges, and extending the period of study in the great majority of schools, no preliminary education is required, and the period of study has in some cases actually been shortened. Only four per cent. of American medical students have literary or scientific degrees, and but two and a-half per cent. of the licensed practitioners are college graduates.

The methods of instruction in the majority of our medical colleges is radically defective. When there is no graded course, as is usually the case, the physician pursuing post-graduate studies, the advanced student and the novice who does not know a vein from an artery, may be found seated in the same lecture-room. The science of medicine has passed the stage when men gave long discourses on the probability of Adam's having had an umbilical cord, or the possibility of spirits existing in a vacuum. A practical training is what is needed; actual contact with disease in hospitals and dispensaries; personal work and research in laboratories, and lectures only to explain relations and weave isolated facts into one harmonious whole, is what is required. This would be far better than the present system of compelling students to listen to lectures five or six hours a day, as if they were expected to absorb the art of medicine from the seats of the lecture-room.

